# Obstructive sleep apnea and periodontitis: a systematic review with considerations on CPAP therapy

**DOI:** 10.2340/aos.v85.46512

**Published:** 2026-07-16

**Authors:** Aida Kukila, Muhammed Manzoor, Aino Salminen, Päivi Mäntylä, Pirkko Pussinen

**Affiliations:** a Institute of Dentistry, University of Eastern Finland, Kuopio, Finland; b Oral and Maxillofacial Diseases, University of Helsinki, Helsinki, Finland

**Keywords:** Periodontitis, obstructive sleep apnea, continuous positive airway pressure, oral microbiome, systematic review

## Abstract

**Objective:**

To evaluate the association between obstructive sleep apnoea (OSA) and periodontitis and to assess the available evidence on the effects of continuous positive airway pressure (CPAP) therapy on periodontal status.

**Materials and methods:**

This review was conducted in accordance with Preferred Reporting Items for Systematic reviews and Meta-Analyses 2020 guidelines. Comprehensive literature searches were performed in Cochrane, PubMed, Scopus, and Web of Science. Study selection and data extraction were carried out independently by two reviewers. A total of 20 original studies met the inclusion criteria. Methodological quality was assessed using the Joanna Briggs Institute critical appraisal tool.

**Results:**

Fourteen of the 20 studies (70%) reported a significant association between OSA and periodontitis. However, only two studies fulfilled all methodological quality criteria, indicating an overall moderate to low quality of evidence. Data on the effects of CPAP therapy on periodontal outcomes were limited, and the available studies generally showed no significant impact.

**Conclusions:**

Current evidence supports an association between OSA and periodontitis, suggesting that OSA may contribute to periodontitis. Robust, well-designed studies with larger sample sizes and rigorous methodology are needed to clarify the nature of this relationship and to determine whether CPAP therapy influences periodontal health.

## Introduction

Periodontitis is a chronic inflammatory disease characterized by periodontal pocket formation, alveolar bone resorption, loss of periodontal attachment, and ultimately tooth loss, driven by the host immune response to dysbiotic microbiota [1]. Obstructive sleep apnea (OSA) is a sleep-related breathing disorder defined by recurrent episodes of complete or partial upper airway obstruction, clinically presenting with snoring, insomnia, excessive daytime sleepiness, hypoventilation, and impaired concentration [2, 3]. During sleep, relaxation of the soft palate and tongue may narrow or occlude the airway, resulting in intermittent reductions or cessation of ventilation lasting for at least 10 seconds [4, 5]. These events lead to hypoxia, hypercapnia, sleep fragmentation, and reduced blood oxygen saturation [2, 6]. Diagnosis is based on polysomnography (PSG), clinical evaluation, and medical history, with severity classified using the Apnea–Hypopnea Index (AHI) as mild (≥ 5 to < 15 events/h), moderate (≥ 15 to < 30 events/h), or severe (≥ 30 events/h) [7]. Intermittent hypoxia may exacerbate systemic inflammation and influence oxidative stress in periodontal tissues, potentially contributing to pathogenesis of periodontitis [8, 9]. OSA is also associated with an increased risk of systemic morbidities [5, 10].

Continuous positive airway pressure (CPAP) is the standard treatment for OSA and is delivered through nasal masks, face masks, nasal prongs, or nasopharyngeal tubes connected to a CPAP device during sleep [11, 12]. CPAP therapy may reduce mouth breathing and xerostomia, particularly when used with heated humidification [12]. Several studies have investigated the relationship between OSA and periodontitis, noting shared risk factors such as age, sex, obesity, diabetes mellitus, smoking, and alcohol consumption, as well as overlapping inflammatory mediators, including interleukin-1β and C-reactive protein (CRP) [3, 13].

This study synthesizes current evidence on the association between OSA and periodontitis and evaluates whether CPAP therapy alters periodontal outcomes, highlighting an important and understudied link between sleep‑related breathing disorders and oral inflammatory disease.

## Materials and methods

**Figure 1:**
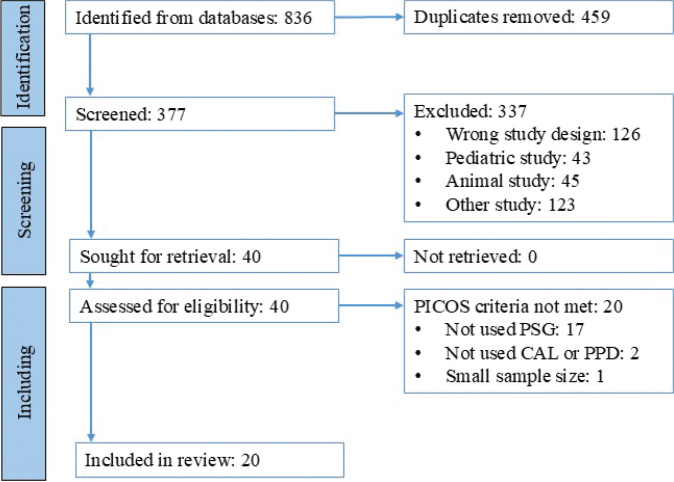
Prisma flow diagram presenting the selection of studies. PICOS: participants, intervention, comparisons, outcomes, and study design; PSG: polysomnography; CAL: clinical attachment level; PPD: probing pocket depth.

The protocol for this systematic review was reported according to the Preferred Reporting Items for Systematic reviews and Meta-Analyses (PRISMA) 2020 statement and checklist. The PRISMA flow diagram was used to illustrate the search results (Figure 1) [14]. This review protocol was registered with the International Prospective Register of Systematic Review (PROSPERO, CRD420251047991).

### Inclusion and exclusion criteria

This systematic review applied the PICOS framework (participants, intervention, comparisons, outcomes, and study design) to determine study eligibility. The PICOS criteria were structured to address the research question: ‘Is there an association between OSA and periodontitis?’ Participants: adults (> 18 years) diagnosed with obstructive sleep apnea and periodontitis.

Intervention: periodontitis diagnosed using clinical attachment level (CAL) and/or probing pocket depth (PPD) and OSA diagnosed using PSG.

Comparisons: adults without OSA and without periodontitis.

Outcomes: any reported association between OSA and periodontitis.

Study design: only original case‑control or cross‑sectional studies with more than 50 participants were included.

Reviews, systematic reviews, editorials, commentaries, opinion pieces, case reports, and non‑English publications were excluded. Studies involving pediatric populations or animal models were also excluded.

### Search methods

A systematic literature review was conducted using the Cochrane Library, PubMed, Scopus, and Web of Science databases up to April 2025. Studies were identified using the Boolean operator AND with the following search terms: ‘periodontitis AND sleep apnea’, ‘microbiome AND sleep apnea’, ‘microbiota AND sleep apnea’, and ‘CPAP AND periodontitis’. This search strategy yielded 836 studies. After applying the predefined exclusion criteria, 40 articles were selected for full-text review. Ultimately, 20 studies met the inclusion criteria and were included in the final analysis (Figure 1). The included studies were published between 2009 and 2024.

### Data collection and analysis

Two reviewers (AK and PP) independently analyzed and evaluated the included studies. Following this independent assessment, they discussed their findings and reached a consensus. Any disagreements were resolved through consultation with the other authors. This process helped mitigate the risk of bias associated with a single reviewer. Additionally, multiple databases were used for data extraction to reduce selection bias. If consensus had not been achieved, further consultation with the other authors would have been undertaken. The data presented in the Results section were collected using a customized Excel spreadsheet (Tables 1 and 2).

**Table 1:** Quality assessment of included case–control studies.

Author	Criteria for inclusion clearly defined?	Study subjects and the setting described in detail?	Exposure measured in a valid and reliable way?	Objective, standard criteria used for measurement of the condition?	Confounding factors identified?	Strategies to deal with confounding factors stated?	Outcomes measured in a reliable way?	Appropriate statistical analysis used?	*n* = yes, out of 8
Arango Jimenez et al., 2023 [10]	Yes	No	Yes	Yes	No	No	Yes	Yes	5
Ashraf et al., 2022 [4]	No	No	Yes	Yes	No	No	Yes	Yes	4
Chen et al., 2023 [8]	Yes	Yes	Yes	Yes	Yes	Yes	Yes	Yes	8
Gamzik-Isik et al., 2017 [3]	Yes	Yes	Yes	Yes	No	No	Yes	Yes	6
Gunaratnam et al., 2009 [16]	Yes	No	Yes	Yes	No	No	Yes	Yes	5
Keller et al., 2013 [17]	No	Yes	Yes	Yes	Yes	Yes	No	Yes	6
Loke et al., 2015 [18]	Yes	Yes	Yes	Yes	Yes	Yes	Yes	Yes	8
Nizam et al., 2014 [19]	No	Yes	Yes	Yes	No	No	Yes	No	4
Nizam et al., 2015 [20]	No	Yes	Yes	Yes	No	No	Yes	Yes	5
Nizam et al., 2016 [21]	No	Yes	Yes	Yes	Yes	No	Yes	No	5
Pico-Orozco et al., 2021 [22]	No	Yes	Yes	Yes	Yes	No	No	No	4
Chen et al., 2021 [30]	Yes	Yes	Yes	Yes	Yes	Yes	No	Yes	7

**Table 2:** Quality assessment of included cross-sectional studies.

Author	Criteria for inclusion clearly defined?	Study subjects and the setting described in detail?	Exposure measured in a valid and reliable way?	Objective, standard criteria used for measurement of the condition?	Confounding factors identified?	Strategies to deal with confounding factors stated?	Outcomes measured in a reliable way?	Appropriate statistical analysis used?	*n* = yes, out of 8
Latorre et al., 2018 [23]	No	No	Yes	Yes	No	No	Yes	Yes	4
Pallavi et al., 2024 [6]	Yes	No	Yes	Yes	No	No	Yes	Yes	7
Seo et al., 2013 [24]	No	Yes	Yes	Yes	No	No	No	Yes	4
Stazić et al., 2022 [25]	No	Yes	Yes	Yes	No	No	Yes	Yes	5
Téllez et al., 2022 [26]	Yes	Yes	Yes	Yes	No	No	Yes	Yes	6
Téllez et al., 2023 [27]	Yes	Yes	Yes	Yes	No	No	Yes	Yes	6
Téllez et al., 2023 [28]	Yes	Yes	Yes	Yes	No	No	Yes	Yes	6
Tranfić Duplančić et al., 2022 [29]	No	Yes	Yes	Yes	No	No	Yes	Yes	6

### The quality assessment

Two reviewers (AK and PP) performed a quality assessment of the included literature using the Joanna Briggs Institute (JBI) critical appraisal tool [15]. We used the checklist for analytical cross-sectional studies that includes questions used for the quality assessment (Tables 1 and 2).

## Results

### Results of the search

Twelve studies were case–control studies, and eight studies were cross-sectional studies [3, 4, 6, 8, 10, 16–30]. One study was from Australia, two from China, five from Colombia, two from Croatia, two from India, one from Korea, one from Spain, one from Taiwan, four from Turkey, and one from the USA.

### Participants and diagnoses

The total sample of human participants was 32,245 subjects. The sample sizes across the studies ranged from 50 to 29,284 participants. The subjects’ age ranged from 18 to >85 years. 17 studies used CAL and PPD to diagnose periodontitis, and three studies used only CAL or PPD [4, 26, 28].

### Type of confounding factors

Confounding factors were identified (Tables 1 and 2) in six of the 20 studies [8, 17, 18, 21, 22, 30]. The most reported confounders included age, body mass index (BMI), diabetes, sex, smoking, and alcohol consumption [8, 18]. Strategies to address confounding were reported in four studies [8, 17, 18, 30]. These strategies involved either adjusting for confounders in the statistical analysis or excluding affected participants from the study [18, 30].

### Data analysis

The quality of the studies was evaluated using a scoring system ranging from one to eight (Tables 1 and 2). Only two studies met all the quality criteria and received the maximum score of eight points [8, 18]. Inclusion criteria were clearly defined in 10 studies [3, 6, 8, 10, 16, 18, 26–28, 30]. Statistical analyses were performed in 17 studies [3, 4, 6, 8, 10, 16–18, 20, 23–30]. Outcomes were measured reliably in 16 studies [3, 4, 6, 8, 10, 16,18–21, 23, 25–29]. Objective and standardized criteria, such as specified diagnostic definitions, were used to define the conditions in all studies. The study populations and settings, including location and study period, were described in detail in 15 studies [3, 8, 17–22, 24, 25–30]. The measurement of exposure was considered valid and reliable across all studies, as the methods were clearly described. In three studies, the control groups did not undergo PSG testing [4, 17, 22], and two studies did not include a control group for comparison [6, 16].

### Outcomes

Fourteen (70%) of the 20 studies reported a significant, direct association between OSA and periodontitis (Tables 3 and 4) [3, 4, 6, 8, 16, 17, 21–27, 30]. Various stages of periodontitis – mild, moderate, and severe – were associated with OSA, while one study specifically reported an association between mild OSA and periodontitis [23]. One study found that disease severity was correlated, with periodontitis being more severe in patients with severe OSA [22]. Associations were also reported between moderate-to-severe OSA and severe periodontitis (stage III–IV) in one study, between severe OSA and stage III periodontitis in two studies, and between severe OSA and severe periodontitis in another [25, 26, 28]. The AHI explained 16.4% of the variability in mean clinical attachment loss (CAL) [25]. According to Pallavi et al., the prevalence of periodontitis increases with the severity of OSA [6]. Reported odds ratios for significant associations between OSA and periodontitis ranged from 1.75 to 4.31 [8, 17, 22, 24].

**Table 3:** Included case–control studies.

Author	Region	Sample size	Age range	Female (%)	Male (%)	Clinical periodontal parameters	Methods of diagnosing OSA	Comparison group	An association between OSA and periodontitis?
Arango Jimenez et al., 2023 [10]	Colombia	60	46–53	50	50	BOP, CAL, GR, PI, PPD	PSG	Yes	No
Ashraf et al., 2022 [4]	India	120	18–77	23.3	76.7	CAL, CPI	PSG	Yes	Yes
Chen et al., 2021 [30]	China	54	25–35	**-**	100	CAL, PPD	PSG	Yes	Yes
Chen et al., 2023 [8]	China	93	24–35	**-**	100	BOP, CAL, PPD	PSG	Yes	Yes
Gamsiz-Isik et al., 2017 [3]	Turkey	163	30–68	25.2	74.9	BOP, CAL, GI, PI, PPD	PSG	Yes	Yes
Gunaratnam et al., 2009 [16]	Australia	66	54.9 ±12.8	18.2	81.8	BOP, CAL, GI, GR, PI, PPD	PSG	No	Yes
Keller et al., 2013 [17]	Taiwan	29,284	18–69+	37.7	62.3	CAL, ICD-9-CM code 523.4, PPD	PSG	Yes	Yes
Loke et al., 2015 [18]	USA	100	28–79	9	91	BOP, CAL, GR, PI, PPD	PSG	Yes	No
Nizam et al., 2014 [19]	Turkey	52	21–64	38.5	61.5	BOP, CAL, PI, PPD	PSG	Yes	No
Nizam et al., 2015 [20]	Turkey	50	21–64	40	60	BOP, CAL, PI, PPD	PSG	Yes	No
Nizam et al., 2016 [21]	Turkey	52	21–64	38.5	61.5	BOP, CAL, PI, PPD	PSG	Yes	Yes
Pico-Orozco et al., 2021 [22]	Spain	114	25–75	50	50	BOP, CAL, CI, PI, PPD	PSG/RP	Yes	Yes

OSA: obstructive sleep apnoea; BOP: bleeding on probing; CAL: clinical attachment loss; CI: calculus index; CPI: community periodontal index; GI: gingival index; GR: gingival recession; PI: plaque index; PPD: probing pocket depth; PSG: polysomnography; RP: respiratory polygraphy.

**Table 4:** Included cross-sectional studies.

Author	Region	Sample size	Age range	Female (%)	Male (%)	Clinical periodontal parameters	Methods of diagnosing OSA	Comparison group	An association between OSA and periodontitis?
Latorre et al., 2018 [23]	Colombia	199	30–85	53.8	46.2	CAL, PPD	PSG	Yes	Yes
Pallavi et al., 2024 [6]	India	500	30–70	50	50	CAL, GI, GR, OHI-S, PPD	PSG	No	Yes
Seo et al., 2013 [24]	Korea	687	47–77	33	67	BOP, CAL, GR, GI, PI, PPD	PSG	Yes	Yes
Stazić et al., 2022 [25]	Croatia	194	35–68	32	68	CAL, FMBS, FMPS, GR, PPD	PSG/PG	Yes	Yes
Téllez et al., 2022 [26]	Colombia	93	30–72	60.2	39.8	BOP, PI, PPD	PSG	Yes	Yes
Téllez et al., 2023 [27]	Colombia	75	>30	53.4	46.6	BOP, CAL, PI, PPD	PSG	Yes	Yes
Téllez et al., 2023 [28]	Colombia	84	30–71	57.1	42.9	BOP, PI, PPD	PSG	Yes	No
Tranfić Duplančić et al., 2022 [29]	Croatia	205	31–51	34.1	65.9	BOP, CAL, GR, PI, PISA, PPD	PSG/PG	Yes	No

OSA: obstructive sleep apnoea; BOP: bleeding on probing; CAL: clinical attachment loss; FMBS: full mouth bleeding score; FMPS: full mouth plaque score; GI gingival index; GR: gingival recession; OHI-S: oral hygiene index-simplified; PI: plaque index: PG: polygraphy; PISA: periodontal inflamed surface area score; PPD: probing pocket depths; PSG: polysomnography.

In addition to clinical parameters, differences between OSA cases and controls were observed in salivary biomarkers, including IL-1β, IL-6, IL-17A, IL-33, apelin, neutrophil elastase, matrix metalloproteinase (MMP)-8, and proMMP-2 [20, 21, 28]. Furthermore, differences in dysbiotic species and bacterial diversity have been reported between OSA patients with and without periodontitis [21, 26–28, 30]. *Candida albicans* and *Prevotella* species have been identified in individuals with both conditions [26].

Two studies evaluated periodontal health status in CPAP users [12, 31]. However, neither met the diagnostic criteria of the present systematic review and were therefore excluded from further analysis. In a population-based case–control study, Carra et al. reported that CPAP/bilevel positive airway pressure (BiPAP) users exhibited similar levels of plaque, calculus, gingival inflammation, as well as comparable numbers of teeth and masticatory units, compared with matched controls [12]. In another study assessing periodontal status 10 years after baseline among individuals without OSA, non-CPAP users, and adherent CPAP users, CPAP therapy was not found to influence periodontal health outcomes [31].

## Discussion

This systematic review indicates that a substantial proportion of the included studies support an association between obstructive sleep apnea (OSA) and periodontitis. Specifically, 14 of the 20 studies (70%) reported a statistically significant association between the two conditions [3, 4, 6, 8, 16, 17, 21–28, 30], suggesting a generally consistent relationship across heterogeneous populations and study designs. This interpretation is further supported by recent Mendelian randomization analyses, which provide genetic evidence consistent with a potential causal effect of OSA on periodontitis risk, while not supporting a reverse effect of periodontitis on OSA [32]. Although earlier literature has proposed a bidirectional relationship, the current body of evidence may suggest a predominantly unidirectional trend from OSA to periodontitis.

A notable limitation of the existing literature is the heterogeneity in diagnostic approaches. Several studies relied on Community Periodontal Index (CPI) scores or registry-based data rather than comprehensive periodontal examinations. While such methods enable the inclusion of large populations, they may reduce diagnostic accuracy and introduce misclassification bias. Similarly, OSA diagnosis varied considerably across studies, ranging from PSG to screening questionnaires such as the Berlin Questionnaire, thereby limiting comparability. Nevertheless, the overall consistency of findings despite these methodological differences strengthens the evidence for an association.

Some studies merit particular attention due to their methodological approaches, although CAL or PPD measurements were necessarily not used. Ytzhaik et al. analyzed data from more than 130,000 individuals using machine learning models and identified both established (age, obesity, and male sex) and novel (periodontal disease and dental restorations) predictors of OSA [5]. Kim et al. confirmed in a population-based cohort a significant relationship between mild to severe chronic periodontitis and OSA [33]. Mi et al. applied Mendelian randomization to demonstrate a potential causal role of OSA in the development of periodontitis [32]. Smoking was inconsistently reported across studies and may have influenced periodontal outcomes in some cohorts [3, 4, 6, 16, 18–25, 29, 34]. Although smoking is a well-established risk factor for periodontitis, its role in OSA appears less pronounced and may be confounded by other factors, such as obesity and comorbid conditions. Furthermore, three studies originated from the same research group and included partially overlapping datasets, which should be taken into account when interpreting the overall weight of evidence [26–28].

Six studies reported no statistically significant association between OSA and periodontitis [10, 18–20, 28, 29]. For example, Loke et al. found no association between OSA and moderate-to-severe periodontitis or other periodontal parameters [18]. Similarly, Nizam et al. observed no differences in salivary or serum inflammatory biomarkers, including MMPs and neutrophil-derived enzymes, between OSA patients and controls [20]. These discrepancies may be explained by differences in study design, sample size, population characteristics, and adjustment for confounding variables.

Systemic inflammation is likely a central mechanism linking OSA and periodontitis. Intermittent hypoxia, a hallmark of OSA, may promote the release of pro-inflammatory cytokines, such as interleukin (IL)-1β, IL-6, IL-17A, and IL-33, in saliva and gingival crevicular fluid, thereby contributing to osteoclastogenesis and periodontal tissue destruction [8, 28]. These cytokines may also stimulate hepatic CRP production, reflecting systemic inflammation [3]. In addition, shared inflammatory pathways may partly explain the co-occurrence of OSA, periodontitis, and systemic conditions such as type 2 diabetes mellitus [35]. Although bidirectional mechanisms have been suggested, current evidence more strongly supports a pathway in which OSA contributes to periodontal disease progression.

Alterations in the oral microbiome may also play a role in the relationship between OSA and periodontitis. Four studies investigated microbial profiles in patients with OSA [21, 26, 28, 30], with findings suggesting an increased prevalence of periodontopathogenic species, including *Prevotella* spp. [26, 30]. Téllez Corral et al. also reported the presence of *Candida albicans* in individuals with both conditions, which may contribute to oral dysbiosis [26]. Factors associated with OSA, such as mouth-breathing and intermittent hypoxia, may impair salivary function and favor microbial shifts that promote periodontal disease progression [19]. These findings suggest that OSA may influence periodontitis through interactions between altered host responses and microbial composition.

Evidence regarding the impact of CPAP therapy on periodontal health remains limited. The available studies generally indicate little or no effect of CPAP on periodontal parameters. Carra et al. found no differences in plaque accumulation, calculus, gingival inflammation, or masticatory function between CPAP or bilevel positive airway pressure users and non-users [12]. Similarly, a long-term follow-up study reported no differences in periodontitis progression among individuals without OSA, untreated OSA patients, and CPAP-adherent patients [31]. Although short-term changes in the oral microbiota have been observed following CPAP use [35], these findings are based on small samples and require confirmation. CPAP therapy may reduce systemic inflammation, as indicated by reductions in CRP levels [36], but its clinical relevance for periodontal outcomes remains unclear.

## Conclusion

Within the limitations of this systematic review, including heterogeneity in study design and diagnostic criteria, the available evidence suggests a significant association between OSA and periodontitis. Current data indicate that OSA may contribute to the development or progression of periodontitis although causality cannot yet be definitively established. Evidence regarding the effect of CPAP therapy on periodontal health is limited and inconclusive.

Further well-designed prospective studies with standardized diagnostic criteria, adequate control of confounding factors, and sufficiently large sample sizes are needed to clarify the nature of this association. In addition, studies incorporating advanced microbiome analyses may provide further insight into the biological mechanisms underlying the relationship between OSA and periodontitis.
